# Iron overload and iron overload-related complications across the clinical spectrum of hemoglobin H disease: a real-world multicenter registry study from Thailand

**DOI:** 10.3389/fmed.2026.1979497

**Published:** 2026-10-28

**Authors:** Sirichai Srichairatanakool, Chattree Hantaweepant, Duantida Songdej, Bunchoo Pongtanakul, Nattiya Teawtrakul, Pornpun Sripornsawan, Napat Laoaroon, Patcharee Komwilaisak, Arunotai Meekaewkunchorn, Pimlak Charoenkwan, Pacharapan Surapolchai, Pranee Sutcharitchan, Noppacharn Uaprasert, Krissana Panrong, Sasinee Hantrakool, Supanun Lauhasurayotin, Kitti Torcharus, Panachai Silpsamrit, Adisak Tantiworawit

**Affiliations:** 1 Division of Hematology, Department of Internal Medicine, Faculty of Medicine, Chiang Mai University, Chiang Mai, Thailand; 2 Thalassemia and Hematology Center, Faculty of Medicine, Chiang Mai University, Chiang Mai, Thailand; 3 Division of Hematology, Department of Medicine, Faculty of Medicine Siriraj Hospital, Mahidol University, Bangkok, Thailand; 4 Division of Hematology and Oncology, Department of Pediatrics, Faculty of Medicine Ramathibodi Hospital, Mahidol University, Bangkok, Thailand; 5 Division of Hematology and Oncology, Department of Pediatrics, Faculty of Medicine Siriraj Hospital, Mahidol University, Bangkok, Thailand; 6 Division of Hematology, Department of Internal Medicine, Srinagarind Hospital, Faculty of Medicine, Khon Kaen University, Khon Kaen, Thailand; 7 Division of Hematology and Oncology, Department of Pediatrics, Faculty of Medicine, Prince of Songkla University, Songkhla, Thailand; 8 Division of Hematology and Oncology, Department of Pediatrics, Faculty of Medicine, Khon Kaen University, Khon Kaen, Thailand; 9 Queen Sirikit National Institute of Child Health, Bangkok, Thailand; 10 Division of Hematology and Oncology, Department of Pediatrics, Faculty of Medicine, Chiang Mai University, Chiang Mai, Thailand; 11 Division of Hematology and Oncology, Department of Pediatrics, Faculty of Medicine, Thammasat University, Pathum Thani, Thailand; 12 Division of Hematology, Department of Internal Medicine, Chulalongkorn University and King Chulalongkorn Memorial Hospital, Bangkok, Thailand; 13 Division of Hematology and Oncology, Department of Pediatrics, Faculty of Medicine, Chulalongkorn University, Bangkok, Thailand; 14 Division of Pediatric Hematology/Oncology, Department of Pediatrics, Phramongkutklao College of Medicine, Bangkok, Thailand; 15 Division of Hematology, Department of Internal Medicine, Phramongkutklao Hospital and College of Medicine, Bangkok, Thailand

**Keywords:** alpha thalassemia, Hb H disease, hemoglobin h disease, iron overload, iron overload complications

## Abstract

**Background:**

Iron overload presents as a major cause of long-term morbidity among thalassemia and is increasingly recognized in patients with hemoglobin (Hb) H disease; however, real-world clinical data defining the burden, complications, and associated factors of iron overload across these patients remain poorly characterized. Understanding these factors is important for optimizing iron surveillance and therapeutic strategies for iron overload in patients with Hb H disease.

**Methods:**

Based on the multicenter registry of Thai patients with Hb H disease, a total of 1,298 patients, comprising both deletional and non-deletional Hb H, were analyzed. Clinical characteristics, iron overload burden, and iron overload-related complications were evaluated across Hb H subgroups. Multivariable regression was used to identify independent predictors of iron overload and any related complications.

**Results:**

Among 1,298 patients with a median age of 17.2 years (IQR 10.0–39.8), 726 patients (56%) had deletional, and 572 (44%) had non-deletional Hb H disease. Patients with non-deletional Hb H had significantly lower hemoglobin and a substantially higher transfusion burden than those with deletional Hb H. Approximately 20% of patients across the clinical spectrum had iron overload, with significantly higher ferritin and liver iron concentration (LIC) in non-deletional Hb H. Additionally, overall iron overload-related complications were significantly higher among non-deletional Hb H (*p* < 0.05). In multivariable analyses, the factors associated with iron overload included non-deletional genotype, increasing age, and Hb E co-inheritance (*p* < 0.01).

**Discussion:**

Iron overload is a significant complication and not uncommon in Hb H disease, particularly among non-deletional subgroups and older patients. These findings emphasize that transfusion burden alone is insufficient to identify patients at risk of iron overload and underscore the need for comprehensive evaluation across the spectrum of Hb H disease, even among non-transfusion-dependent thalassemia (NTDT). To our knowledge, this study represents one of the largest multicenter real-world evaluations of iron overload and its clinical consequences specifically in Hb H disease.

## Introduction

Alpha (*α*)-thalassemia is one of the most common inherited hemoglobin disorders worldwide, particularly in Southeast Asian, the Middle East, and among Mediterranean populations (1, 2). Thailand has one of the highest prevalences of *α*-thalassemia in Southeast Asia, with the carrier rate estimated at approximately 20%–30% of the population (3). Among *α*-thalassemia cases, hemoglobin (Hb) H disease is one of the most common clinically significant subtypes, resulting from the compound heterozygosity of *α*^0^-thalassemia deletion with either single *α*-globin gene deletion (deletional Hb H disease) or non-deletional point mutation (non-deletional Hb H disease) (4). Clinical manifestations of individuals with Hb H disease are remarkably diverse, and the patients may exhibit a wide spectrum of disease severity and thalassemia-related complications (5). Several studies have increasingly recognized that significant morbidities, including iron overload-related complications, are not uncommon in patients with Hb H disease, particularly in the non-deletional subtype (6–8).

While the impact of iron overload in *β*-thalassemia has been extensively reported, its significance and related complications in patients with *α*-thalassemia remain less clearly defined. Individuals with Hb H disease, particularly those with the non-deletional subtype, may develop profound anemia that requires regular transfusions, manifesting as transfusion-dependent thalassemia (TDT) phenotype, thereby resulting in secondary iron overload. Nevertheless, significant iron overload may occur even in patients with non-transfusion-dependent thalassemia (NTDT), typically beyond the first decade of life (9, 10). Ultimately, the resulting iron overload can contribute to various complications, including cardiac dysfunction, liver disease, infection, and multiple endocrinopathies (11). Currently, guideline recommendations have been largely extrapolated from the data of *β*-thalassemia subjects, which may not have accurately reflected the pattern of iron accumulation in patients with *α*-thalassemia, particularly among those with the NTDT phenotype (12, 13). To address these knowledge gaps, we have conducted a comprehensive analysis of the data obtained from a multicenter *α*-thalassemia registry. This study aimed to determine the characteristics of iron overload and its complications, as well as factors associated with iron overload, thereby providing real-world evidence to guide management strategies for *α*-thalassemia patients.

## Methods

### Study design and patients

This study analyzed data obtained from the nationwide, multicenter registry of patients with Hb H disease in Thailand, established by the Red Blood Cell Disorders and Aplastic Anemia Committee with additional support from the Thai Society of Hematology (TSH). All participants were recruited from 15 hematology centers across Thailand, including both pediatric (nine units) and adult (six units) hematology divisions, from December 2022 to December 2023. This study was approved by the institutional review boards of all participating centers, in accordance with the Declaration of Helsinki. Informed consent was waived as the study involved no more than minimal risk to participants.

Eligible participants included both pediatric and adult patients diagnosed with Hb H disease, confirmed by high-performance liquid chromatography or capillary electrophoresis, with DNA analysis performed when indicated. Clinical data were collected, including thalassemia diagnosis, history of splenectomy, relevant blood transfusion history, and iron overload. NTDT and TDT phenotypes were classified according to transfusion burden and disease severity based on the established international recommendation (13, 14).

### Laboratory investigations and iron assessments

Laboratory data included complete blood count (CBC), basic laboratory investigations, and serum ferritin levels. These data were collected during routine clinical care rather than at a single predefined time point. Hb levels and serum ferritin levels were reported as the mean of the available measurements obtained during the 12-month inclusion period, which may better reflect overall clinical status than a single laboratory assessment. As a real-world registry, the timing and frequency of laboratory assessments varied according to clinical practice and among participating centers. Notably, the ferritin levels obtained during documented active infection or inflammatory conditions were excluded from analyses. Magnetic resonance imaging (MRI) for the determination of liver iron concentration (LIC) was performed by the local institute, and the most recent available result was recorded together with the corresponding serum ferritin at the time of MRI. The decision to perform MRI was primarily based on clinical considerations and resource availability. All investigations were performed using the standard techniques described by the manufacturers.

### Iron overload and iron overload-related complications

In accordance with the Thalassemia International Federation (TIF) recommendation (13, 14), serum ferritin levels ≥800 ng/mL for NTDT, or ≥1,000 ng/mL for TDT, were classified as iron overload and met the threshold for initial iron chelation. Additionally, patients with serum ferritin ≥2,500 ng/mL were defined as severe iron overload. Based on MRI results, the patients with LIC >5 (for TDT) or >7 (for NTDT) mg Fe/g dry weight (dw) were considered to have iron overload, while the patients who had LIC >15 mg Fe/g dw were defined as severe iron overload. Clinical complications related to iron overload were systematically assessed, including adrenal insufficiency, diabetes mellitus, hypogonadism, hypothyroidism, and infection, as shown in Supplementary Table S1 (15–18).

### Statistical analysis

Statistical analyses were performed using STATA version 18.0 (StataCorp, College Station, TX, USA). Continuous variables were presented as mean ± standard deviation (SD) or median values with interquartile range (IQR), while categorical variables were demonstrated as counts and percentages. Kaplan–Meier curves were used to estimate the cumulative incidence of complications over age, with time origin defined as the date of birth, and the event was defined as the date on which the complication was first documented during the observation period. Cox proportional hazards models were used to calculate crude hazard ratios (HR) for time-to-event outcomes. Multivariable regression analysis was performed to identify factors associated with iron overload. A *p*-value of <0.05 was considered statistically significant.

## Results

### Patient characteristics

From December 2022 to December 2023, a total of 1,298 patients with Hb H disease were included. These patients were then separated accordingly, 726 (56%) with deletional Hb H and 572 (44%) with non-deletional Hb H disease. The median age in this cohort was 17.2 years (IQR 10.0−39.8), while deletional Hb H patients were significantly older than those with non-deletional Hb H (20.2 years vs. 15.4 years, *p* < 0.001). 17.3% of patients had co-inheritance of Hb H disease with Hb E mutations, along with other less common combinations, including *β*-thalassemia trait and *β*-thalassemia/Hb E disease.

Most patients were classified as NTDT (84.7%), while the remaining required regular blood transfusions. Non-deletional Hb H disease had significantly lower mean Hb levels (8.5 ± 1.4 g/dL vs. 9.6 ± 4.8 g/dL, *p* < 0.001) and required more regular transfusions when compared with those with deletional Hb H (30.8% vs. 3.0%, *p* < 0.001). Additionally, the age at first transfusion was substantially younger (median 4.0 years vs. 10.8 years, *p* < 0.001) in the non-deletional group. Splenectomy had been performed in 56 patients (4.3%), which was significantly higher in those with non-deletional Hb H disease when compared with deletional Hb H (8.4% vs. 1.1%, *p* < 0.001), as is shown in Table 1.

**Table 1:** Baseline characteristics of the enrolled patients with hemoglobin H disease.

Characteristics	Total (*n* = 1,298)	Deletional Hb H (*n* = 726)	Non-deletional Hb H (*n* =5 72)	*p*-value
Age				
▪ Age (years), median (IQR)	17.2 (10.0–39.8)	20.2 (10.5–52.1)	15.4 (9.1–27.8)	<0.001
Co-inheritance of *β*-thalassemia, n (%)				
▪ None	1,023 (79.4)	617 (85.6)	406 (71.6)	
▪ Heterozygous Hb E	204 (15.8)	71 (9.8)	133 (23.5)	
▪ Homozygous Hb E	20 (1.6)	6 (0.8)	14 (2.5)	
▪ Heterozygous β-thalassemia (β-thalassemia trait)	3 (0.2)	1 (0.1)	2 (0.4)	
▪ β-thalassemia/Hb E disease	4 (0.3)	2 (0.3)	2 (0.4)	
▪ Others	13 (1.0)	10 (1.4)	3 (0.5)	
▪ Unknown	21 (1.6)	14 (1.9)	7 (1.2)	
Current disease status, n (%)				
▪ TDT	198 (15.3)	22 (3.0)	176 (30.8)	<0.001
▪ NTDT	1,100 (84.7)	704 (97.0)	396 (69.2)	
Splenectomy, n (%)				
▪ Yes	56 (4.3)	8 (1.1)	48 (8.4)	<0.001
▪ No	1,242 (95.7)	718 (98.9)	524 (91.6)	
Complete blood count				
▪ Hb level at baseline, mean ± SD^1^	9.1 ± 3.7	9.56 ± 4.8	8.51 ± 1.4	<0.001
▪ MCV (fL), mean ± SD	61.8 ± 9.9	58.9 ± 8.9	67.2 ± 9.3	<0.001
▪ MCH (pg), mean ± SD	17.9 ± 2.8	17.4 ± 2.8	18.8 ± 2.5	<0.001
▪ MCHC (g/dL), mean ± SD	29.2 ± 8.3	29.5 ± 1.9	28.6 ± 13.7	0.068
▪ RDW (%), mean ± SD	25.7 ± 15.9	25.2 ± 13.3	26.6 ± 19.7	0.190
Blood transfusion history				
Age at first transfusion (years), median (IQR)	5.4 (1.7–16.5)	10.8 (2.9–47.6)	4.0 (1.2–10.0)	<0.001
Age at initial regular transfusion (years), median (IQR)	4.6 (1.2–9.2)	8.0 (3.5–48.8)	4.3 (1.1–8.6)	0.007
Reason for regular transfusion, n (%)				
▪ Low hemoglobin levels	135 (10.4)	13 (1.8)	122 (21.3)	<0.001
▪ Limited daily activities	19 (1.5)	5 (0.7)	14 (2.4)	0.010
▪ Spleen enlargement	37 (2.9)	1 (0.1)	36 (6.3)	<0.001
▪ Growth failure	45 (3.5)	5 (0.7)	40 (7.0)	<0.001
▪ Bone change	21 (1.6)	3 (0.4)	18 (3.1)	<0.001

Hb, hemoglobin; HSCT, hematopoietic stem cell transplantation; IQR, interquartile range; MCH, mean corpuscular hemoglobin; MCHC, mean corpuscular hemoglobin concentration; MCV, mean corpuscular volume; NTDT, non-transfusion-dependent thalassemia; RDW, red cell distribution width; SD, standard deviation; TDT, transfusion-dependent thalassemia.

### Characteristics of iron overload and iron chelation therapy

Approximately 20% of the patients experienced iron overload, of which the iron burden was significantly higher in patients with non-deletional Hb H, with median serum ferritin levels of 589.0 ng/mL (IQR 267.3–1179.5), as compared to 201.0 ng/mL (IQR 83.0–570.0) in the deletional Hb H subgroup (*p* < 0.001). 173 (30.2%) patients with non-deletional Hb H met the threshold for initial treatment with iron chelation according to their serum ferritin levels.

Unfortunately, only 190 patients (14.6%) were evaluated with the MRI assessment in our study, comprising 48 and 142 patients with deletion and non-deletional Hb H, respectively. In contrast to using serum ferritin, approximately half of the patients in our cohort met the indication for iron chelation using the LIC criteria. Severe iron overload more frequently occurred in patients with non-deletional Hb H disease when compared with deletional Hb H, where the criteria were defined by serum ferritin levels or LIC (Table 2).

**Table 2:** Iron overload and iron chelation therapy of the study population.

Characteristics	Total (*n* = 1,298)		Deletional Hb H (*n* = 726)		Non-deletional Hb H (*n* = 572)		*p*-value
Age group, n (%)							
▪ < 10 years	323 (24.9)		163 (22.5)		160 (28.0)		<0.001
▪ 10 to 20 years	395 (30.4)		197 (27.1)		198 (34.6)		
▪ > 20 years	580 (44.7)		366 (50.4)		214 (37.4)		
Mean serum ferritin in 12 months, (ng/mL)							
▪ Serum ferritin in 12 months, median (IQR)	360 (119.6–891.8)		201 (83.0–570.0)		589 (267.3–1179.5)		<0.001
	NTDT	TDT	NTDT	TDT	NTDT	TDT	
	250.0 (103.6–595.0	1,229.0 (845.0–1,824.0)	185.6 (81.6–521.3)	1,486.0 (1,012.0–2,822.9)	382.4 (180.8–729.5)	1,205.2 (826.5–1,791.6)	
Mean serum ferritin (ng/mL), n (%)							
▪ < 300 for NTDT < 500 for TDT	493 (46.2)		343 (59.7)		150 (30.5)		<0.001
▪ 300 to <800 for NTDT 500 to <1,000 for TDT	305 (28.6)		136 (23.7)		169 (34.3)		
▪ 800 to <2,500 for NTDT 1,000 to <2,500 for TDT	225 (21.1)		81 (14.1)		144 (29.3)		
▪ ≥2,500	44 (4.1)		15 (2.6)		29 (5.9)		
LIC (mgFe/g dw), n (%)^2^							
▪ ≤5	98 (51.6)		32 (66.7)		66 (46.5)		0.004
▪ >5 to 15	74 (38.9)		16 (33.3)		58 (40.8)		
▪ >15	18 (9.5)		0 (0.0)		18 (12.7)		
Current iron chelation therapy, n (%)							
▪ None	985 (75.9)		647 (89.1)		338 (59.1)		<0.001
▪ Yes	313 (24.1)		79 (10.9)		234 (40.9)		
Types of iron chelator (≥1 iron chelator), n (%)							
▪ DFO	30 (2.3)		5 (0.7)		25 (4.4)		<0.001
▪ DFP	232 (17.9)		53 (7.3)		179 (31.3)		<0.001
▪ DFX-DT	98 (7.6)		11 (1.5)		87 (15.2)		<0.001
▪ DFX-FCT	5 (0.4)		0 (0.0)		5 (0.9)		0.016
▪ Others	2 (0.2)		2 (0.3)		0 (0.0)		0.510
Doses of iron chelator (mg/day), mean ± SD							
▪ DFO	1,500 ± 591.6		1,000 ± 707.11		1,611.11 ± 546.5		0.201
▪ DFP	2,746.4 ± 1,397.8		2,270.50 ± 1,421.8		2,940 ± 1,345.1		0.002
▪ DFX-DT	804.4 ± 424.5		791.7 ± 541.8		806.7 ± 404.4		0.911
▪ DFX-FCT	NA		NA		NA		NA

DFO, deferoxamine; DFP, deferiprone; DFX-DT, deferasirox-dispersible tab; DFX-FCT, deferasirox-film-coated tablet; Hb, hemoglobin; IQR, interquartile range; LIC, liver iron concentration; NTDT, non-transfusion-dependent thalassemia; SD, standard deviation; TDT, transfusion-dependent thalassemia.

Although three patients (2.6%) with the NTDT phenotype under the age of 20 years demonstrated evidence of iron overload, the prevalence of iron overload progressively increased in those aged ≥20 years. Conversely, among 113 patients with TDT, iron overload could occur earlier, even in patients aged <10 years. Additionally, severe iron overload was more common in TDT and could even appear in patients younger than 20 years (Supplementary Figure 1A). Characterized by Hb H subgroups, 76 patients (53.5%) with the non-deletional genotype exhibited iron overload, with 18 individuals (12.7%) having severe iron overload. On the other hand, 16 patients (33.3%) with the deletional genotype had increased LIC, whereas none of the patients developed severe iron overload defined by LIC criteria (Supplementary Figure 1B).

Iron chelation therapy was more significantly prescribed in non-deletional Hb H disease (40.9%) when compared with deletional Hb H (10.9%), *p* < 0.001. Deferiprone (DFP) was the most frequently used iron chelator, prescribed in 31.3% of non-deletional and 7.3% of deletional Hb H cases, whereas deferasirox (DFX) and deferoxamine (DFO) were infrequently used in our cohort. Furthermore, the mean DFP dose was also significantly higher in subjects with non-deletional Hb H disease (Table 2).

### Incidence of iron overload-related complications

The cumulative incidence of iron overload-related complications has gradually increased over time (Figure 1A). These complications included adrenal insufficiency, diabetes mellitus, hypogonadism, hypothyroidism, and infection. The results also demonstrated that non-deletional Hb H disease significantly increased the risk of iron overload-related complications when compared with deletional Hb H disease [crude hazard ratio [crude HR] 2.79, 95% confidence interval [CI]; 1.73–4.51, *p* < 0.001], as illustrated in Figure 1B. The patients with combined Hb H disease and Hb E mutations demonstrated a trend of increased overall iron overload-related complications when compared with patients with isolated Hb H disease; however, no statistical significance was defined (*p* = 0.16), as presented in Supplementary Figure S2.

**Figure 1:**
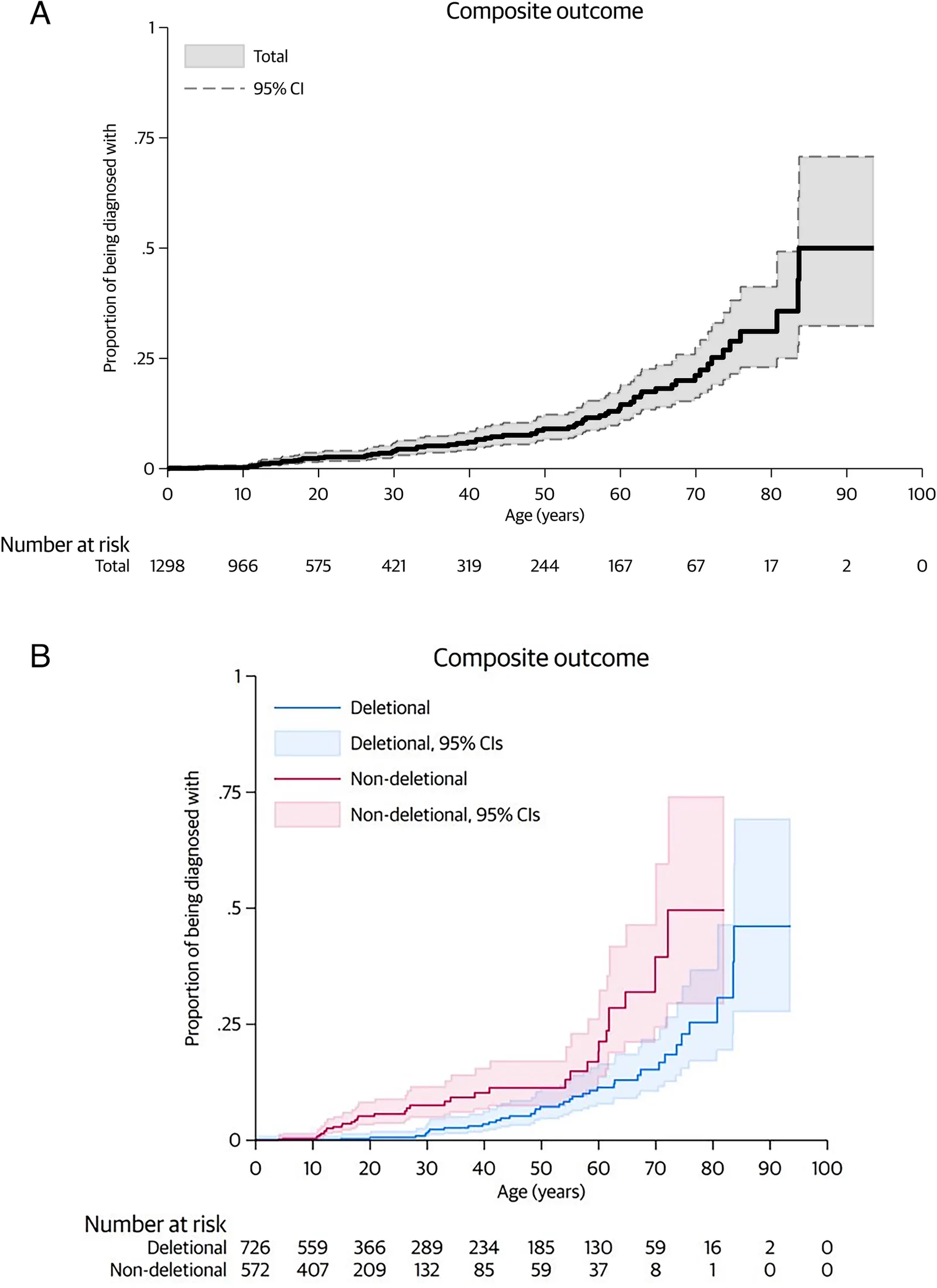
**(A)** cumulative incidence of all iron overload-related complications (composite outcome) in all included patients with hb H disease. **(B)** Cumulative incidence of all iron overload-related complications (composite outcome) between deletional and non-deletional Hb H disease.

According to organ-specific complications from iron overload (Figure 2), the analyses revealed significantly increased risks of complications in non-deletional Hb H disease when compared with the deletional Hb H subtype. These complications included adrenal insufficiency (crude HR 12.49, 95% CI; 1.52–102.46, *p* = 0.019) and infections (crude HR 4.35, 95% CI; 1.58–12.01, *p* = 0.004). However, the risks of hypogonadism, hypothyroidism, and diabetes mellitus were not statistically significant between the subgroups. The median age of occurrence of adrenal insufficiency and infection was 12.76 years (IQR 11.38–30.04) and 28.64 years (IQR 21.85–40.50), respectively (Data not shown).

**Figure 2:**
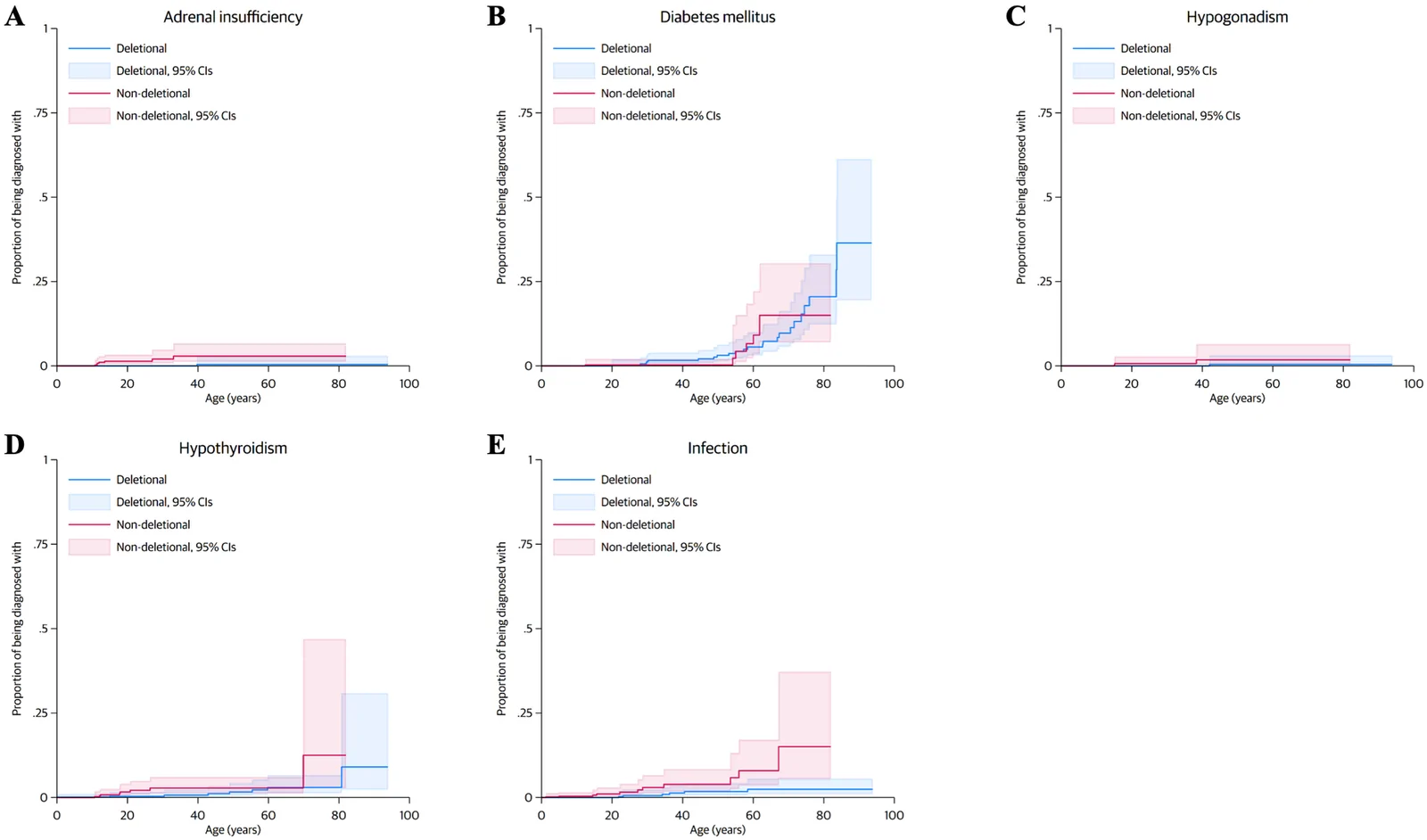
Cumulative incidence of iron overload-related complications (each defined organ-related complication) between deletional and non-deletional hb H disease. **(A)** Adrenal insufficiency. **(B)** Diabetes mellitus. **(C)** Hypogonadism. **(D)** Hypothyroidism. **(E)** Infection.

### Factors associated with the development of iron overload

As shown in Table 3. The independent significant risk factors associated with the development of iron overload were the non-deletional Hb H genotype [adjusted odds ratio (aOR) 1.79, 95% CI 1.40–2.28, *p* < 0.001], the age of the patient along with each increasing decade (aOR 1.10, 95% CI 1.04–1.16, *p* < 0.001), and co-inheritance with Hb E mutations (aOR 1.85, 95% CI 1.37–2.51, *p* < 0.001). However, gender and splenectomy did not significantly associate with the risk of iron overload in our study.

**Table 3:** Multivariable analysis of factors associated with iron overload (mean 12-month ferritin >800 ng/mL for NTDT and >1,000 ng/mL for TDT), *N* = 1,254.

Variables	Multivariable analysis	
	Adjusted OR (95%CI)	*p*-value
Hb H disease		
• Deletional Hb H	**(Reference)**	
• Non-deletional Hb H	1.79 (1.40, 2.28)	<0.001
Age (each 10 years increase)	1.10 (1.04, 1.16)	<0.001
Gender		
• Female	**(Reference)**	
• Male	1.09 (0.86, 1.37)	0.481
Co-inheritance		
• None	**(Reference)**	
• Heterozygous or Homozygous Hb E	1.85 (1.37, 2.51)	<0.001
• Heterozygous β-thalassemia	3.46 (0.31, 39.12)	0.315
• β-thalassemia/Hb E	5.78 (0.58, 57.49)	0.134
Splenectomy status		
• No splenectomy	**(Reference)**	
• Splenectomy	0.70 (0.39, 1.25)	0.224

## Discussion

This nationwide, multicenter registry represents one of the largest real-world cohorts of patients with Hb H disease, providing evidence that iron overload is clinically significant and not uncommon in Hb H disease. This study demonstrated that the clinical spectrum of iron overload markedly diverged between deletional and non-deletional genotypes, reflecting the clinical heterogeneity of *α*-thalassemia syndromes determined by underlying mutations and disease modifiers (8).

Our study demonstrated that patients with non-deletional Hb H disease were significantly younger, had lower Hb levels, experienced greater transfusion burdens, and had higher numbers of splenectomies when compared with deletional Hb H, thus emphasizing the more severe phenotype associated with non-deletional Hb H. These findings align with prior reports, which found that the phenotypes of non-deletional Hb H disease, most commonly Hb H-Constant Spring (CS) disease, are usually more severe than those with deletional Hb H disease (10, 19, 20). This severity arises from the production of unstable *α*-globin variants, such as Hb CS, together with the intrinsic Hb H instability, which contributes to further ineffective erythropoiesis (IE) and hemolysis, resulting in more pronounced clinical manifestations. Conversely, most patients with deletional Hb H have only mild phenotypes and rarely need frequent transfusions (1, 5). Among patients with deletional Hb H disease in our study, predominantly those with SEA deletion *α*-thalassemia 1 with 3.7 kb deletion *α*-thalassemia 2 (–SEA/-*α*^3^·⁷) and SEA deletion *α*-thalassemia 1 with 4.2 kb deletion *α*-thalassemia 2 (–SEA/-*α*⁴·^2^), there were no significant differences in clinical phenotypes, consistent with a previous large cohort study (21).

Up to one-fourth of patients, especially those with non-deletional Hb H disease, met the criteria for starting iron chelation. The findings were consistent with historical studies which found that many adult patients with Hb H disease will have some degree of iron overload, particularly those with the non-deletional Hb H subtype (2, 21–23). Iron overload in Hb H disease is multifactorial and varies according to transfusion status. In patients with the NTDT phenotype, IE increases erythropoietic drive and promotes erythroferrone-mediated suppression of hepcidin production, thereby enhancing intestinal iron absorption and promoting progressive iron accumulation despite the absence of regular transfusions (24, 25). In contrast, transfusional iron loading represents a predominant mechanism of iron accumulation in patients with the TDT phenotype (11, 14). Therefore, transfusion history alone may not accurately reflect iron burden in Hb H disease, particularly among patients with non-deletional Hb H.

Notably, roughly half of all patients with Hb H disease in our study meet the indication for iron chelation using the LIC criteria, suggesting that ferritin may underestimate tissue iron, particularly in patients with severe IE and high erythropoietic activity (26). The mechanism of iron overload in TDT appears to be the same between *α*- and *β*-thalassemia. However, the rate of iron accumulation in the NTDT phenotype, as well as in those with *α*-thalassemia, is much slower than that observed in TDT and *β*-thalassemia, respectively (24–27). Consistent with a greater degree of IE, a previous report of *β*-thalassemia intermedia demonstrated that a substantial proportion of patients aged 10 years or older exhibited iron overload (28). On the other hand, the patients with Hb H disease in our study generally encountered a substantial iron burden by the age of ≥20 years. However, the patients with non-deletional Hb H subgroup appeared to be at substantial risk of early-onset iron overload, even before the first decade of life. These findings further support the need for early assessment and regular monitoring of iron burden in patients with Hb H disease, particularly those with the non-deletional subtype, preferably using MRI-based assessment, regardless of transfusion history (9, 10).

The number of patients receiving iron chelation therapy and the intensity of chelation were significantly higher in patients with non-deletional Hb H disease, with deferiprone being the most widely used as an iron chelator in our cohort. This was due to nationally established policies. Although there are differing approved iron chelators in first-line treatment among different countries, all three iron chelators have been well established for their efficacy (24, 29, 30).

In our study, the iron overload-related complications illustrated a progressive, time-dependent increase in incidence among overall patients with Hb H disease, underscoring the nature of iron-mediated organ damage that steadily increases with advancing age (25). The cumulative incidence of iron overload-related complications was significantly higher in patients with non-deletional Hb H disease, together with an earlier onset of complications. Interestingly, the combination of non-deletional Hb H disease and Hb E mutations demonstrated higher iron overload-related complications when compared with isolated Hb H disease; however, no statistically significant differences were observed. This was possibly due to the small patient subgroups.

Among the specific complications, adrenal insufficiency and infection were significantly higher in non-deletional Hb H. In contrast, hypogonadism and hypothyroidism were found to be more frequent but did not reach statistical significance. Endocrinopathies in our study were slightly different from a Chinese cohort (6), with up to 84% and 13% of patients having hypogonadism and hypothyroidism, respectively. It is worth noting that the baseline hemoglobin levels of the study were lower when compared with our cohort, possibly leading to increased iron absorption and resulting in a higher incidence of iron overload-related complications. There was no statistically significant difference in diabetes incidence between Hb H genotypes, consistent with those of a previous study (31). Compared to *β*-thalassemia, the onset of iron overload-related complications in patients with *α*-thalassemia was literally slower. In our study, the median age at which most iron overload-related complications occur is beyond 20 years, indicating a relatively later onset when compared with *β*-thalassemia, in which the complications typically develop after a decade of life (32). Nevertheless, an earlier onset of complications was still observed in some patients with Hb H disease, particularly among patients with the TDT phenotype and those with non-deletional Hb H. Interestingly, adrenal insufficiency demonstrated an earlier age of onset among the assessed complications, with a comparable pattern reported in *β*-thalassemia patients (15). These findings underscore the importance of individualized and early organ-specific screening, especially adrenal insufficiency, in *α*-thalassemia patients.

Although the MRI-based method measuring cardiac T2* was not assessed in this study, a previous study demonstrated that a significant impact of cardiovascular complications was associated with a transfusion-dependent status (33). This may be explained by the different dynamics of iron loading between TDT and NTDT, wherein iron absorption is predominantly increased in the NTDT phenotype, with preferential iron accumulation in the liver leading to elevated LIC, whereas cardiac iron deposition is less prominent. These findings have also been observed in patients with Hb H disease, in whom cardiovascular complications appear to occur at a very low frequency, particularly among patients with the NTDT phenotype (34, 35).

We identified independent factors associated with iron overload in patients with Hb H disease, including genotype, age, and co-inheritance of the Hb E mutation. The non-deletional Hb H genotype had a substantially higher iron burden than the deletional subtypes, which has been largely reported in previous studies (5, 9, 11). Interestingly, patients with Hb H disease, who had a combined Hb E mutation, further experienced iron overload and its related complications. The co-inheritance of *α*- and *β*-thalassemia has been previously acknowledged as ameliorating disease severity due to a reduction in globin imbalance (36). However, a recent study reported that non-deletional Hb H disease with the co-inherited Hb E may have a more severe clinical phenotype than those without Hb E co-inheritance, with lower baseline Hb levels reported (37).

The mechanisms underlying the more severe phenotypes associated with Hb E co-inheritance, particularly in non-deletional Hb H disease, remain incompletely understood. The proposed explanation is that the interaction of unstable *α*-globin variants, such as Hb CS, with *β*^E^ chains may further exacerbate IE in non-deletional Hb H disease. In addition, the *β*^E^ tetramers themselves might be unstable, potentially leading to decreased red blood cell survival and increased hemolysis (37). These effects may collectively contribute to greater clinical severity and iron burden observed in patients with this genotype combination.

As previously mentioned, the burden of iron overload will gradually progress in parallel with increasing age. Those outcomes are consistent with our findings, demonstrating that the risk of iron overload significantly increased with age in each subsequent decade. These findings suggest that patients with significant risk factors, regardless of transfusion burden, may have a higher prevalence of iron overload and its related complications (38, 39). Accordingly, regular iron overload evaluations, particularly in patients with established risk factors, are essential even in NTDT patients.

The association between increased serum ferritin levels and LIC has been previously observed in patients with non-transfusion-dependent Hb H disease (40). Unfortunately, the correlation between ferritin and LIC in our study demonstrated only a weak correlation with iron burden (Supplementary Figure S3). These results indicate the limitations of iron overload assessment using serum ferritin measurement in patients with Hb H disease. This was especially true in those with NTDT, whose serum ferritin was generally lower than those with TDT at equivalent LIC levels (41). For this reason, MRIs remain the gold standard for iron overload assessment, offering a precise evaluation of tissue iron deposition. Notably, serum ferritin still provides a practical surrogate in routine care for assessing iron overload burden and is useful in limited resource settings (9, 42).

The major limitation of our study was the restricted availability of MRI data, as it was only available for 14.6% of the patients. This limitation may have been due to the limited available resources. Importantly, the age of patients for MRI assessments, as recommended by TIF, advised initiating MRI for iron overload after the age of 10 years (12). Additionally, key outcomes, including cardiac T2* measurements and hepatic complications such as hepatitis and cirrhosis, recognized as major comorbidities in NTDT, were not available in our study. However, a prospective cohort demonstrated that abnormal liver function occurred in approximately 10% of patients with Hb H disease (34), highlighting the potential hepatic involvement in this population. Nevertheless, no cases of hepatocellular carcinoma were reported in our cohort. The cross-sectional design could also be considered a limitation in terms of evaluating the dynamics and efficacy of iron chelation in our study. Further longitudinal research is warranted to better characterize the effects of iron chelation therapy. Lastly, the potential for referral bias should be mentioned, as patients with more severe phenotypes may be more frequently referred to tertiary care centers, while individuals with milder forms may be underrepresented.

In conclusion, this large multicenter registry provides real-world evidence demonstrating that iron overload is a clinically significant and potentially underrecognized condition in Hb H disease, particularly in non-deletional genotypes. Moreover, progressive iron accumulation with increasing age and defined risk factors may consequently lead to various complications. These findings indicate that transfusion burden alone is insufficient to identify the risk of iron overload and emphasize the early detection of iron overload and the initiation of iron chelation therapy for the prevention of organ dysfunction and improvement of long-term outcomes in patients with Hb H disease.

## Data Availability

Data are available upon reasonable request from the corresponding authors.

## Footnotes

^1^Hemoglobin (Hb) level at baseline was defined as Hb level measured during a steady period, without an acute hemolytic episode.

^2^The number of patients underwent MRI assessment in our study were 190 patients (14.6%), comprising 48 deletional Hb H disease and 142 non-deletional Hb H disease.

## Supplementary material

SUPPLEMENTARY FIGURE S1 Proportion of patients (deletional vs non-deletional Hb H disease) with liver iron concentration among defined age groups.

SUPPLEMENTARY FIGURE S2 Cumulative incidence of all iron overload-related complications between Hb H disease with Hb E combination and Hb H disease without Hb E combination.

SUPPLEMENTARY FIGURE S3 The correlation between ferritin within 3 months of MRI date and liver iron concentration in NTDT and TDT patients.
