# Supplementary material for: Iron overload and iron overload-related complications across the clinical spectrum of hemoglobin H disease: a real-world multicenter registry study from Thailand
Source: Front Med (Lausanne). 2026 Oct 28;13:1979497. doi: 10.3389/fmed.2026.1979497 (PMC13751424; doi:10.3389/fmed.2026.1979497)
Supplement: Supplementary file 1 [file Datasheet1.pdf]

Supplementary Table 1: The definition of clinical complications

**Complications**	**Definition / Criteria**
Adrenal insufficiency	Measured serum cortisol levels during glucagon stimulation test (GST) <18 µg/dL[12]
Diabetes mellitus	Fasting blood sugar ≥126 mg/dL, or 2-hour postprandial blood sugar ≥200 mg/dL, or the presence of hyperglycemic symptoms and a random plasma glucose ≥200 mg/dL[13]
Hypogonadism	Females: >13 yrs, not yet Tanner B2 or >14 yrs requiring estrogen replacement therapy or >15 yrs with primary amenorrhea<br>Males: >14 yrs, not yet Tanner G2 or on androgen replacement therapy or >17 yrs, not yet Tanner G4[14]
Hypothyroidism	TSH >4.7 µU/L and a free T_4 <0.8 ng/dL[15]
Infection	Clinically significant infectious event requiring hospitalization and/or antimicrobial therapy, with or without microbiological confirmation
