# Supplementary material for: Iron overload and iron overload-related complications across the clinical spectrum of hemoglobin H disease: a real-world multicenter registry study from Thailand
Source: Front Med (Lausanne). 2026 Oct 28;13:1979497. doi: 10.3389/fmed.2026.1979497 (PMC13751424; doi:10.3389/fmed.2026.1979497)
Supplement: Supplementary file 2 [file Datasheet2.pdf]

Proportion of patients (NTDT vs TDT) with liver iron concentration among age groups

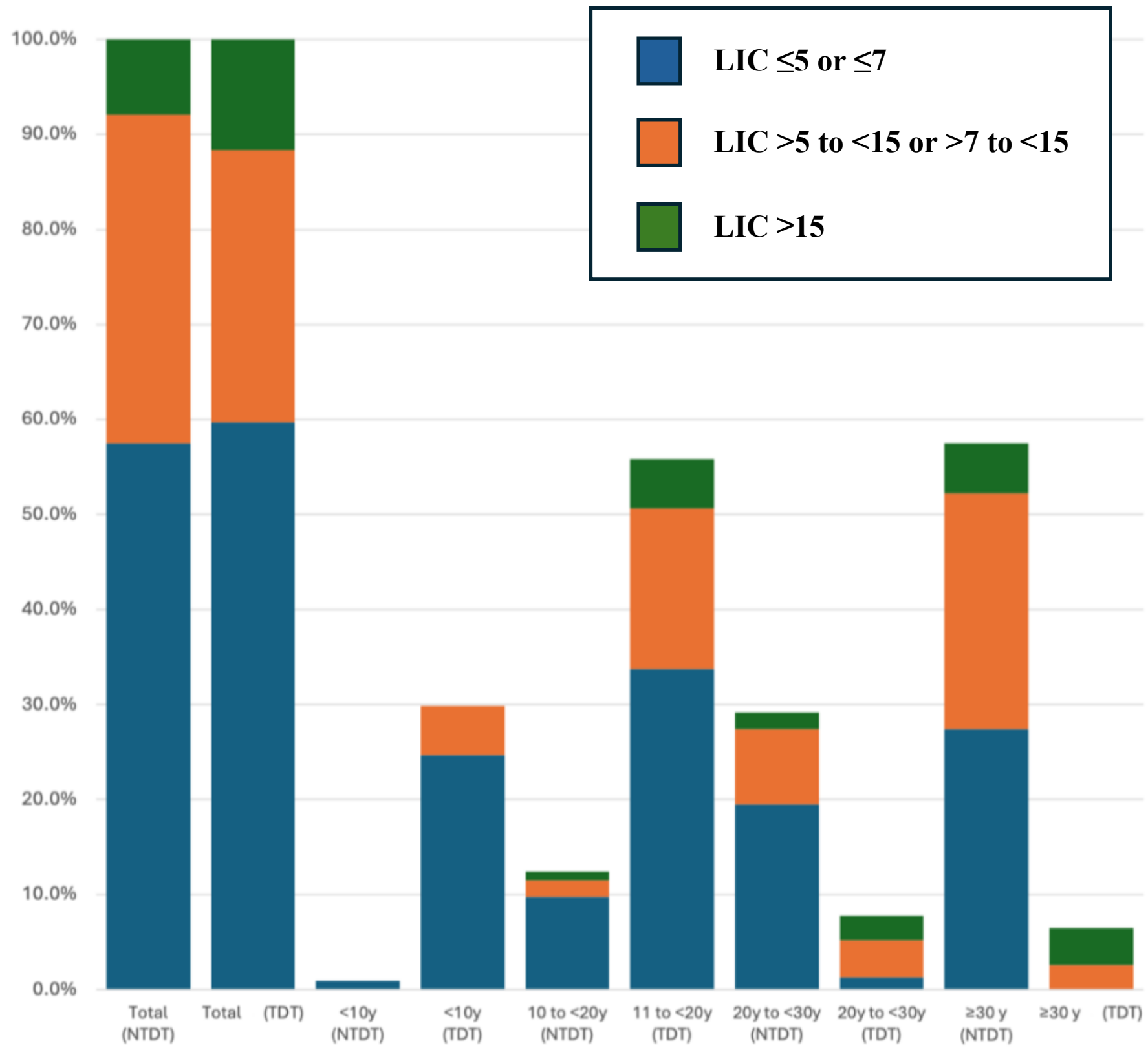

Liver iron concentration	≤ 5 or ≤ 7	> 5 or 7 to <15	≥ 15	Total
▪ Total (NTDT)	57.5% (N = 65)	34.5% (N = 39)	8.0% (N = 9)	100.0% (N = 113)
▪ Total (TDT)	59.7% (N = 46)	28.6% (N = 22)	11.7% (N = 9)	100.0% (N = 77)
▪ < 10 yr (NTDT)	0.9% (N = 1)	0.0% (N = 0)	0.0% (N = 0)	0.9% (N = 1)
▪ < 10 yr (TDT)	24.7% (N = 19)	5.2% (N =4)	0.0% (N = 0)	29.9% (N = 23)
▪ 10 to < 20 yr (NTDT)	9.7% (N = 11)	1.8% (N = 2)	0.9% (N = 1)	12.4% (N = 14)
▪ 11 to < 20 yr (TDT)	33.8% (N = 26)	16.9% (N = 13)	5.2% (N = 4)	55.8% (N = 43)
▪ 20 yr to < 30 yr (NTDT)	19.5% (N = 22)	8.0% (N = 9)	1.8% (N = 2)	29.2% (N = 33)
▪ 20 yr to < 30 yr (TDT)	1.3% (N = 1)	3.9% (N = 3)	2.6% (N = 2)	7.8% (N = 6)
▪ ≥ 30 yr (NTDT)	27.4% (N = 31)	24.8% (N = 28)	5.3% (N = 6)	57.5% (N = 65)
▪ ≥ 30 yr (TDT)	0.0% (N = 0)	2.6% (N = 2)	3.9% (N = 3)	6.5% (N = 5)
